# Effect of three modalities on emergence agitation among post-traumatic stress disorder patients undergoing laparoscopy: a randomized controlled study

**DOI:** 10.1186/s12888-024-05525-5

**Published:** 2024-01-29

**Authors:** Heba Ahmed Abdelaziz, Yomna E. Dean, Ahmed Mohamed Ahmed Elshafie

**Affiliations:** 1 Lecturer of Mental Health, Department of Family Health, Alexandria High Institute of Public Health, Alexandria, Egypt; 2 Lecturer of Anesthesia and Surgical Intensive Care, Department of Anesthesia and Surgical Intensive Care, Alexandria University, Faculty of Medicine, Alexandria, Egypt; 3 Alexandria Medical Center (AMC), Alexandria, Egypt

**Keywords:** PTSD, Ketamine, Breathing, Anesthesia, Emergence agitation

## Abstract

**Background and aim:**

Emergence agitation (EA) after general anesthesia is common in patients with post-traumatic stress disorder (PTSD). Due to the recent worldwide events such as the Covid-19 pandemic and wars, PTSD is not rare. Accordingly, a reliable, cost-effective anesthetic protocol to lower the incidence of EA is crucial. Therefore, we aimed to compare three different interventions for avoiding EA in PTSD patients undergoing gynecological laparoscopic surgery. Participants were divided into four groups: 1: performing pre-operative relaxation techniques (deep breathing exercise and progressive muscle relaxation [PMR]); 2: administrating intra-operative Ketamine; 3: applying both previously mentioned strategies and 4 as controls.

**Methods:**

This study was carried out on 144 adult women scheduled for gynecological laparoscopy, randomly allocated into four groups: three intervention groups and a control group (36 each). Women aged 18-45 years old, with a diagnosis of PTSD were included in the study. Patients with a positive history of major neurological, cardiovascular, metabolic, respiratory, or renal disease were excluded. Any patient who reported the use of psychiatric drugs were also excluded from the study. Data was analyzed using IBM SPSS Statistics software version 26. Kolmogorov- Smirnov was used to verify the normality of the distribution of variables. Odds ratio was calculated to clarify the strength and direction of the association between intervention groups and control. Data was deemed significant at a *p*-value ≤0.05.

**Results:**

Heart rate (HR) and Mean Arterial Blood Pressure (MABP) intra-operative and post-operative till 24 hours were significantly lower in groups 1, 2, and 3 compared to group 4 (*p<*0.001). There was a significant statistical difference in the intraoperative HR percentage decrease. MABP percentage decrease post-operative was higher in all the intervention groups with no statistically significant difference, except for group 1 compared to group 4, which was statistically significant (12.28 ± 11.77 and 6.10 ± 7.24, *p=*0.025). Visual Analogue Scale measurements were significantly less in the intervention groups 1, 2, and 3 compared to group 4. On Riker sedation–agitation scores, group 1 was 85 times more likely to be non-agitated (85 (15.938 – 453.307), *p<*0.001), group 2 was 175 times more likely to be non-agitated (175 (19.932–1536.448), *p<*0.001) and group 3 was protected against agitation.

**Conclusion:**

Pre-operative relaxation techniques (breathing exercises and PMR) significantly lowered HR, MABP, VAS score, and EA than controls. These effects were not significantly different from intra-operative ketamine injection or the combination of both (relaxation techniques and ketamine). We recommend routine pre-operative screening for PTSD and the application of relaxation techniques (breathing exercises and PMR) in the pre-operative preparation protocol of PTSD-positive cases as well as routine practical application of preoperative relaxation techniques. Further studies on using pre-operative relaxation techniques in general could be cost-effective.

## Introduction

Emergence agitation/delirium (EA or ED) carries a high risk for both patients and staff in the operating theatre [1]. EA is reported in 5% to 10% of general surgery patients of all ages. It is an acute condition during early recovery from general anesthesia, where the patient may show confusion, hallucinations, or delusions. That may be manifested as restlessness, involuntary physical activity, disorientation, excitation, and thrashing [2].

Although the mechanism of EA remains unclear, the risk factors include age (more in children or older adults), male sex, use of inhalational anesthetics with low blood–gas partition coefficients, anticholinergic drugs, premedication with benzodiazepines, full urinary bladder, postoperative pain, and the presence of invasive devices (e.g., nasal tube or urinary catheter). The operation itself might be a risk factor according to its type, pre-operative preparation, and duration of surgery (more in longer operations) [3].

Generally, EA subsides spontaneously, yet it sometimes has significant consequences, such as injury to the patient, staff, falling, or bleeding at the surgical site due to excessive movement, accidental removal of drains or tubes, or respiratory depression. In addition, the medical staff could be injured, and materials could be damaged, leading to increased medical care costs [3].

Individuals having post-traumatic stress disorder (PTSD) were found to be more liable to EA [1].

EA among individuals who suffered from PTSD was a topic of interest for researchers, especially since pre-existing PTSD independently predicted the frequency of EA [4] and was associated with resistance to regular postoperative ways of emergence from anesthesia [5].

PTSD is a disabling psychiatric disorder that follows a traumatic experience involving a threat to one’s own life or physical integrity and is associated with witnessing death, injury, or threat to the physical integrity of another person. It is associated with high degrees of disability with functional and cognitive impairment [6].

After a traumatic event, an individual with PTSD experiences symptoms for one month or more. PTSD symptoms could include recurrent, unpleasant, distressing memories/dreams or intrusive thoughts, nightmares, and flashbacks that may be accompanied by persistently re-experiencing the traumatic event. The patient develops hyperarousal symptoms, sleep disturbance, and avoidance of stimuli associated with the trauma, with a physiological reaction to being exposed to the traumatic reminders. PTSD also involves negative cognitive or mood alterations leading to intense sadness and guilt feeling [7].

It was estimated that the lifetime prevalence of PTSD globally among the trauma-exposed ranged from 0.5% to 14.5%. Trauma varies in type, response, social support, and endogenous factors of individuals [8].

General anesthesia affects the brain’s amygdalocentric neurocircuit (AN) and alters the function between the hippocampus, amygdala, and medial prefrontal cortex (responsible for amnesia during anesthesia and decreasing hyperarousal). PTSD also makes biological changes in the AN in the brain, causing excessive uncontrolled fear to be transmitted through the AN to the brainstem and hypothalamic regions. These changes increase the potential for hyperarousal and agitation, affecting the emergence from general anesthesia [5].

During emergence from anesthesia, auditory sensation (of sounds in the operating room) is the first to be regained, followed by tactile and nociceptive stimulation. These stimuli increase the activity of the amygdala (through N-methyl-D-aspartate [NMDA] receptor activation), which is already under-regulated in PTSD and, therefore, less inhibited by the medial prefrontal cortex and hippocampus. Signals from the hippocampus pass to the hypothalamus and then to periaqueductal gray matter, increasing motor activity by stimulating neurons in the brainstem and spinal cord, ending in excessive movement and uncooperative behavior, indicating EA [9].

Ketamine is a dissociative analgesic that unexpectedly proved beneficial in managing EA when applied early during the operation. Its mechanism is not fully explained, but it might be attributed to antagonizing glutamate NMDA receptors. Ketamine antagonizes Glutamate, which forms intrusive memories and decreases fear response from NMDA amygdala activation. It has a further hypnotic action through binding to Hyperpolarization-Activated Cation Channel (HCN1) hyperpolarization-activated channels. The analgesic effect of ketamine is also significant for the management of EA symptoms [5].

Relaxation techniques have been one of the most frequently and successfully identified in PTSD symptom alleviation [10, 11].

Deep breathing exercises [12] and progressive muscle contraction/relaxation technique (PMR) [13], in general, have been found effective in stimulating parasympathetic relaxation and reducing anxiety. Accordingly, they decrease heart rate and negative emotion, improve sleep quality, and promote neuromuscular relaxation [14], specifically in pre-operative patients before surgery [12–14].

The bi-directional cortical influences between the central and autonomic nervous systems explained the physiological action of relaxation exercises. The Central Autonomic Network (CAN) allows the goal-directed behavior areas in the brain to modulate the viscera. These areas are mostly limbic and include the insula, anterior cingulate cortex (ACC), amygdala, and hippocampus. The neurovisceral integration model extends to the prefrontal cortex, where their influence eventually affects the heart rate and initiates endocrine responses through vagal nerve stimulation (VN) [15]. The VN has long been known as a two-way communication between the brain and the heart during emotion. Cognitive and emotional components influence the vagal high frequency (HF) component of heart rate variability. Interestingly, CAN also works bottom-up, where VN afferent modular termini projections reach limbic and cortical regions, affecting cognitive control and leading to more body relaxation [16].

A good anesthetic plan generally considers avoiding excessive pain and adverse stimuli during emergence, reducing EA, and adjusting the onset and duration of medications. Patients who suffer from PTSD are more prone to EA and could benefit from complementary therapy [5].

Anesthetic staff can make use of screening for PTSD perioperatively, which can provide standardized multidisciplinary guidelines for the best use of intraoperative medications, pain control, emergence agitation, and other possible complications, with a specific protocol in cases with PTSD [1, 5].

In this context, the current study is a trial to compare the effect of three different interventions in avoiding EA in PTSD patients, which are namely, pre-operative application of relaxation techniques (specifically breathing exercise and PMR), early intra-operative ketamine injection, and applying both relaxation techniques and ketamine.

### Aim of the work

#### General objective

This research aims to compare three different intervention techniques used for avoiding emergence agitation in post-traumatic stress disorder (PTSD) patients undergoing gynecological laparoscopic surgery.

### Specific objectives

Assess the outcome of different strategies on anesthesia emergence, namely, performing pre-operative relaxation techniques (deep breathing exercise and progressive muscle contraction-relaxation [PMR]), administrating intra-operative ketamine, and applying both previously mentioned strategies together.

## Method

### Study setting

The present study was conducted in Alexandria, El-Shatby University Hospital, Egypt.

### Study design

A randomized control trial was used to conduct the study.

### Target population

The study was carried out on 144 adult women between the ages of (18-45) years, divided into four groups: three intervention groups and a control group (36 each). All of the studied female patients belong to the American Society of Anesthesiologists (ASA) grade I or II and are scheduled for gynecological laparoscopy for benign pathologies under general anesthesia. The sample size was calculated based on effect size 0.5, according to Gupta et al. 2016 [17] and Epi-info version 7 software with 5% marginal error and 95% confidence interval based on 18% prevalence of emergence agitation among PTSD cases (but the study was on children) [18] and 6.66 odds ratio among PTSD adult cases [4].

### Type of sample and method of selection

A team of anesthesiologists and nurses in the operative theatre in Alexandria El-Shatby hospital was trained on the application of the Arabic version of Post-Traumatic Stress Disorder (PTSD) Checklist, civilian version "PCL-C" [19] for screening of PTSD, any female screening positive (above the cutoff score of 50) was subjected to one of three techniques as follows; relaxation exercise by the nurse 10-15 minutes before the anesthesia, ketamine 0.5 mg/Kg intra-operative or both techniques.

### Randomization

All female participants scheduled for gynecological laparoscopy for benign pathologies under general anesthesia were screened for PTSD. Only those who screened positive were randomly allocated into one of four groups using the computerized block randomization technique [20] after a full explanation of the aim of the study and obtaining their consent.

Allocation numbers were saved in opaque envelopes with an intern resident (who was not involved in the study). After screening of the females, those who were illegible received one of the three interventions except for the cases allocated as controls, according to their allocation upon opening the envelope.

Blinding was done by masking the type of intervention from the patients and the statistician. The researchers were blinded regarding which cases received which intervention, and so were the statisticians. The cases who received ketamine were blinded, while the cases who received breathing exercises could not conclude if they also received ketamine or not [21].

### Inclusion criteria

- Women in the reproductive age (18- 45 years old).
- Scheduled for gynecological laparoscopy for benign pathologies.
- Positive screening for post-traumatic stress disorder.

### Exclusion criteria

Patients were excluded from the study if they:

- Have a positive medical history of pelvic inflammatory disease associated or not associated with chronic pelvic pain.
- Have major neurological, cardiovascular, metabolic, respiratory, or renal disease.
- Have a history of chronic abdominal pain.
- Using psychiatric drugs.
- Have a history of comorbidities that may have an influence on postoperative nausea and vomiting (PONV) like diseases.

### Recruitment and categorization of patients

Recruitment of the patients was done by the consultant gynecologist involved in the study from El-Shatby University Hospital between January 2023 and August 2023. The mental health consultant was involved in the preoperative screening and PTSD data collection through a predesigned structured questionnaire as well as training and supervision of relaxation techniques application (breathing exercises and PMR) by the staff who demonstrated them preoperatively to the assigned groups of patients. The anesthesia consultant's role included preoperative assessment, intraoperative induction, and maintenance of anesthesia, including administration of ketamine, supervision of postoperative recovery, and measurement and management of EA whenever needed.

### Preoperative evaluation and preparation

- o Proper history taking and clinical examination.
- o Routine laboratory investigations.
- o Screening for Post-Traumatic Stress Disorder was conducted through the application of the Arabic version of the PTSD Checklist, the civilian version "PCL-C" [22, 23].

The PCL-C consists of 17 items that correspond to the DSM-III-R symptoms of PTSD. Examinees are instructed to indicate how much they have been bothered by each symptom in the past month using a 5-point (1-5) scale. The anchors for the severity ratings range from "Not at all" to "Extremely." PCL-C applies to any traumatic event. The PCL is an easily administered self-report rating scale for assessing the 17 DSM-III-R symptoms of PTSD. The PCL has excellent test-retest reliability. Internal consistency is very high for each of the three items corresponding to the DSM-III-R symptom clusters and the full 17-item scale. The PCL can be used as a continuous measure of PTSD symptom severity by summing scores across the 17 items. The optimally efficient cutoff score was 50 [22, 23]. Those who tested positive for PTSD were recruited, a full explanation of the aim of the study was made clear, confidentiality and willingness for participation were assured, and oral consent was taken from those who participated.

- Using computerized blocked randomization, participants were divided into four groups, as follows:
  1. Group 1: the first intervention group who received preoperative relaxation techniques (breathing exercises and PMR).
  2. Group 2: the second intervention group administered intra-operative ketamine.
  3. Group 3: the third intervention group who were exposed to both preoperative relaxation techniques and intra-operative ketamine.
  4. Group 4: a control group who did not receive any intervention.
- Participants who were allocated to perform the preoperative relaxation techniques (groups 1 and 3) were guided through the exercises:
  1. Breathing exercise: with the eyes closed, inhale a deep breath through the nose, counting from 1 to 4 or 5, then exhale through the mouth, slowly counting from 1 to 6 or more. After that, normal breaths are taken, then the deep breathing exercise is repeated up to 10 times.
  2. Progressive muscle contraction/ relaxation (PMR): systematically contracting then relaxing specific muscle groups progressively from up-down the body. Starting with the neck, then shoulders, upper arms, forearms, wrists, hands and fingers, chest, abdomen, thighs, legs, ankles, feet, and toes. Muscle contraction should be gentle (mild to moderate) and performed simultaneously with inhalation, followed by relaxation, which occurs concurrently with long exhalation (with a similar pattern of breathing to that of breathing exercise).

### In the preoperative holding area

- All patients were placed in a rest supine position, and a large 18 gauge antecubital IV line was inserted to allow blood sampling without a tourniquet.
- Then, all patients received premedication with 0.1 mg/kg midazolam intramuscularly 30 minutes before induction of anesthesia.

### Anesthetic technique

- Upon arrival in the operating room, all standard monitors were applied.
- The anesthesia technique was standardized in all the groups. All patients received general anesthesia; after preoxygenation with 100% oxygen, induction of anesthesia was done using propofol 2-3 mg/kg, 0.6 mg/kg rocuronium, fentanyl 1 mcg/kg,

Participants who were allocated to take intra-operative ketamine (groups 2 and 3) received 0.5 mg/Kg intravenous ketamine just after induction of anesthesia (using propofol 2-3 mg/kg, 0.6 mg/kg rocuronium, fentanyl 1 mcg/kg)

- Tracheal intubation was applied using the appropriate size of ETT after 2 minutes of mask ventilation. Anesthesia was maintained with isoflurane. Mechanical ventilation was maintained to keep end-expiratory CO2 partial pressure between 34 and 40 mmHg.
- All laparoscopic procedures were performed by the same highly experienced gynecologist, using the same instrumentation, and the pneumoperitoneum will be maintained constant at 12 mmHg.
- During surgery, arterial blood pressure, heart rate, oxygen saturation, and ETCO2 were continuously monitored.
- Before skin closure, 1 g paracetamol was given by IV infusion for analgesia.
- At the end of the surgery, inhalational anesthesia was stopped, residual neuromuscular block was antagonized with atropine (0.01mg.kg-1) and neostigmine (0.04 mg.kg-1), and then patients were extubated.
- In the postoperative period, all patients received ketorolac 30mg/8 hours.

### Measurements

The following parameters were measured:

1. Demographic data: Age, weight, height, and duration of surgery.
2. Vital signs (i.e.): Heart rate (beat per minute) and mean arterial blood pressure (MABP) in mmHg were recorded before insufflation of the abdomen, every 15-minute intraoperative, immediately postoperative, and every 4 hours for the rest 24 postoperative hours constituting the study period.
3. Pain assessment was conducted using the "Visual analogue scale" [24]:

The pain was assessed by the direct marking on the visual analog scale (VAS), which consisted of a 10 cm line, 0 cm equivalent to no pain, and 10 cm denoting the worst imaginable pain. Patients were asked to indicate on the line where the pain is in relation to the two extremes.

**Table:** 

Modified Aldrete Score		
Criteria	Ability	Score
Activity	Ability to move voluntarily or on command	
	Four extremities	2
	Two extremities	1
	No movement	0
Respiration	Ability to cough and breathe deeply freely	2
	Dyspnea, shallow or limited breathing	1
	Apnea	0
Circulation	Blood pressure within 20 mm Hg of pre-sedation level	2
	Blood pressure within 20-50 mm Hg of pre-sedation level	1
	Blood pressure within +50 mm Hg of pre-sedation level	0
Consciousness	Fully awake	2
	Arousable on calling	1
	Not responding	0
Oxygen (O_2_) Saturation	Able to maintain O_2_ saturation > 92% on room air	2
	Need O_2_ to maintain O_2_ saturation > 90%	1
	O_2_ saturation is < 90% even with O_2_ supplementation	0

- Visual analogue scale was recorded immediately postoperatively, then after 1, 2, and 3 and every 6 hours for the rest of 24 postoperative hours constituting the study period.
- Rescue analgesia in the form of nalbuphine (4 mg) was given IV when VAS score is ≥ 4 at any time post-operatively in the first 24 hours.

4) Recovery from anesthesia was assessed using “Modified Aldert’s score” [25].

The score indicates recovery from anesthesia and detects any delayed emergence, with the maximum total score being 10; a score of at least 9 is required for discharge from the post-anesthesia care unit (PACU).

5) Emergence agitation:

Measured according to Riker sedation–agitation scores, where emergence agitation is defined as a score ≥5 [26].

**Table:** 

**Score**	**Description**	**Definition**
7	Dangerous agitation	Pulling at endotracheal tube. Tries to remove catheters, climb over bedrail, strike at staff, and/or thrashing side-to-side.
6	Very agitated	Does not calm despite frequent verbal reminding of limits. Requires physical restraints. Bites endotracheal tube.
5	Agitated	Anxious or mildly agitated. Attempts to sit up. Calms with verbal instructions.
4	Calm and co-operative	Calm, awakens easily, and follows commands.
3	Sedated	Difficult to arouse. Awakens to verbal stimuli or gentle shaking, but drifts off again. Follows simple commands.
2	Very sedated	Arouses to physical stimuli. Does not communicate or follow commands. May move spontaneously.
1	Unarousable	Minimal to no response to noxious stimuli.

6) Postoperative complications

Any postoperative-related complication occurring at any time in the 24 hours of the study was spotted, recorded, and treated accordingly, such as:

1. Postoperative nausea and vomiting (PONV). Intravenous metoclopramide (10 mg) and ondansetron (4 mg) were given IV as the first and second lines for treating nausea and vomiting, respectively.
2. Hemodynamic instability (blood pressure ≤ 20 % of baseline, tachycardia ≥100 beats/minute, and bradycardia <60 beats/minute).
3. Arrhythmias.

### Statistical analysis

Collected data were statistically analyzed in IBM SPSS Statistics version 26, using the appropriate techniques to achieve the aim of the study. The Kolmogorov- Smirnov was used to verify the normality of the distribution of variables. Comparisons between different groups of independent samples were assessed using the Kruskal-Wallis test. Comparisons between groups for categorical variables were measured using the Wilcoxon Signed-Rank test, Marginal homogeneity test, and Chi-square test (Fisher or Monte Carlo). Pairwise comparisons between each 2 groups were done using the Post Hoc Test (Dunn's for multiple comparisons test). Odds ratio was calculated to clarify the strength and direction of the association between intervention groups and control. Pearson coefficient correlated between two normally distributed quantitative variables. The significance of the obtained results was judged at the 5% level (*p ≤*0.05).

## Results

Table 1 shows that all the subjects included in the study were female patients with Post-Traumatic Stress Disorder (PTSD) undergoing gynecological laparoscopic surgery, who were matched for age, weight, and duration of surgery.

**Table 1:** Demographic characteristics, post-traumatic stress disorder checklist, civilian version “PCL-C” and duration of surgery among female patients undergoing gynecological laparoscopic surgery

**Demographic data (Mean ± SD.)**	**Group 1** **(No.**^**a**^**= 36)**	**Group 2** **(No. = 36)**	**Group 3** **(No. = 36)**	**Group 4** **(No. = 36)**	**Test of Sig.**	*p*
**Age**	33.64 ± 7.77	31.78 ± 6.92	30.72 ± 6.84	30.61 ± 5.63	*F=*1.518	0.212
**Weight**	74.83 ± 14.02	71.94 ± 11.07	71.81 ± 12	74.58 ± 12.58	*F=*0.623	0.601
**PTSD total Score**^**b**^	57.28 ± 1.61	57.19 ± 1.72	57 ± 2.97	57.92 ± 1.83	*F=*1.282	0.283
**Duration of surgery in minutes**						
Mean ± SD.	56.11 ± 16.86	54.61 ± 10.82	53.72 ± 8.15	56.78 ± 8.06	H=3.546	0.315
Median (Min. – Max.)	50.0 (30.0 – 95.0)	52.50 (40.0 – 90.0)	52.50 (40.0 – 75.0)	55.0 (45.0 – 75.0)		

Group 1: Relaxation technique (breathing and muscle contraction/relaxation exercises)

Group 2: Ketamine intravenous injection

Group 3: Relaxation technique and intravenous Ketamine injection

Group 4: Control group

*SD* Standard deviation, *F* F for One Way ANOVA test, *H* H for Kruskal Wallis test, *χ*^*2*^ Chi square test, *MC* Monte Carlo, *p p* value for comparing between the studied four groups

^a^*No* number

^b^*PTSD* Post Traumatic Stress Disorder

**Fig. 1:**
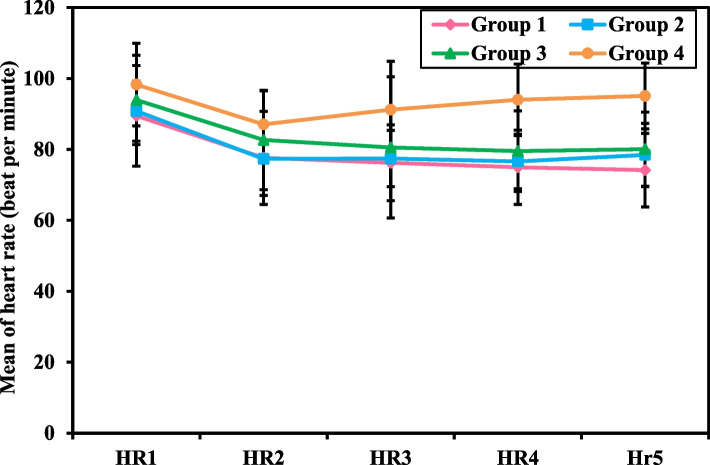
Intra-Operative Heart Rate (HR) Measurements of Female Patients Undergoing Gynecological Laparoscopic Surgery

**Fig. 2:**
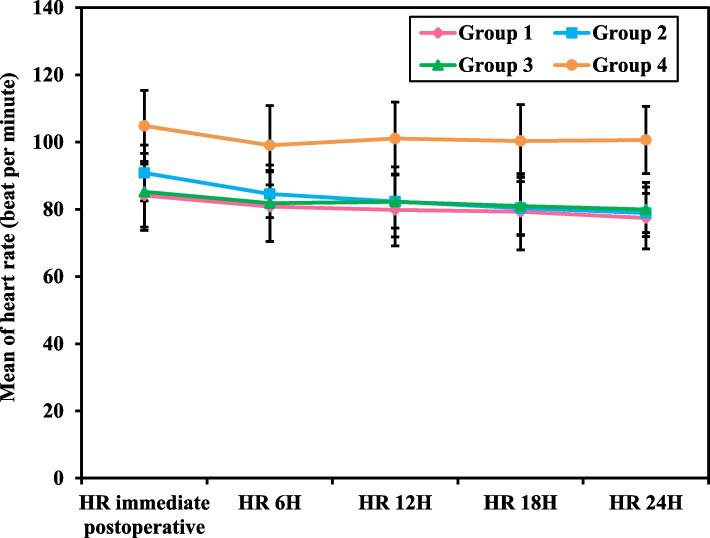
Post-Operative Heart Rate (HR) Measurements of Female Patients Undergoing Gynecological Laparoscopic Surgery

Table 2, Figs. 1 and 2 represent the Heart Rate (HR), which was measured five times intraoperatively every 15 minutes (HR 1 -5), as well as the heart rate that was measured five times postoperatively, starting immediate postoperative, after 6, 12, 18 and till 24 hours postoperative (HR IP, HR 6h, HR 12h, HR 18h and HR 24h). HR (1-5) and HR (IP to HR 24h) were all significantly lower in groups 1, 2, and 3 compared to group 4 (*p<*0.001). There was a significant statistical difference in the intraoperative percentage decrease from the first intra-operative measurement (HR1) consistently till the 5th (HR5), which was 16.19 ± 10.87, 13.47 ± 7.70, and 14.41 ± 7.38 beats per minute among the intervention groups 1, 2 and 3 respectively, while it was 2.69 ± 8.38 in the control group (*p<* 0.001). On the other hand, there was no significant difference in the percentage decrease of HR1 to HR5 between each intervention group and the other. However, a significantly higher percentage decrease was found from the HR IP till HR 24h among group 2 compared to 1, 3, and 4 (*p=*0.036, *p=* 0.001, and *p<*0.001, respectively).

**Table 2:** Intra- and post-operative Heart Rate (HR) measurements of female patients undergoing gynecological laparoscopic surgery

**Heart Rate (HR)** **(in beats per minute)**	**Group 1** **(No. = 36)**	**Group 2** **(No. = 36)**	**Group 3** **(No. = 36)**	**Group 4** **(No. = 36)**	**F**	*p*
**Intra-operative HR**						
**HR1 (Basal HR)**	89.47^a^ ± 14.21	90.81^a^ ± 8.46	93.94 ± 12.61	98.28 ± 11.66	3.878^c^	0.011^c^
**HR2 (after 15 m.**^d^**)**	77.64^a^ ± 13.13	77.33^a^ ± 10.28	82.67 ± 13.99	87.06 ± 9.43	5.464^c^	0.001^c^
**HR3 (after 30 m.)**	76.22^a^ ± 10.68	77.42^a^ ± 7.96	80.56^a^ ± 19.90	91.22 ± 13.64	8.841^c^	<0.001^c^
**HR4 (after 45 m.)**	74.97^a^ ± 10.48	76.61^a^ ± 7.68	79.53^a^ ± 11.35	94.00 ± 10.09	27.216^c^	<0.001^c^
**HR5 (after 60 m.)**	74.17^a^ ± 10.36	78.44^a^ ± 8.92	80.08^a^ ± 10.45	95.08 ± 9.30	31.213^c^	<0.001^c^
**Percent Decrease from HR 1 – 5**	**16.19 ± 10.87**	**13.47 ± 7.70**	**14.41 ± 7.38**	**2.69 ± 8.38**	**17.765**^c^	<0.001^c^
**Significance between groups**	**p**_**1**_**=0.549, p**_**2**_**=0.822, p**_**3**_**<0.001**^c^**, p**_**4**_**=0.968, p**_**5**_**<0.001**^c^**, p**_**6**_**<0.001**^c^					
**HR Immediate Postoperative (IP) till after 24 hours**						
**HR IP**	84.11^a^ ± 9.38	90.83^a^ ± 8.30	85.22^a^ ± 11.46	104.83 ± 10.55	32.680^c^	<0.001^c^
**HR 6H**^**e**^	80.81^a^ ± 10.33	84.56^a^ ± 7.02	81.81^a^ ± 11.33	99.06 ± 11.79	24.475^c^	<0.001^c^
**HR 12H**	79.83^a^ ± 10.67	82.33^a^ ± 7.90	82.22^a^ ± 10.43	101.06 ± 10.82	34.840^c^	<0.001^c^
**HR 18H**	79.28^a^ ± 11.32	80.36^a^ ± 7.91	80.97^a^ ± 8.74	100.33 ± 10.82	38.182^c^	<0.001^c^
**HR 24H**	77.42^a^ ± 9.21	78.92^a^ ± 5.83	79.94^a^ ± 8.09	100.64 ± 10.01	61.145^c^	<0.001^c^
**Percent Decrease from HR IP – 24H**	**7.69 ± 7.70**	**12.62 ± 8.19**	**5.64 ± 5.84**	**3.57 ± 8.70**	9.189^c^	<0.001^c^
**Significance between groups**	**p**_**1**_**=0.036**^c^**, p**_**2**_**=0.670, p**_**3**_**=0.108, p**_**4**_**=0.001**^c^**, p**_**5**_**<0.001**^c^**, p**_**6**_**=0.663**					

Data was expressed in mean ± SD

Group 1: Relaxation technique (breathing and muscle contraction/relaxation exercises)

Group 2: Ketamine intravenous injection

Group 3: Relaxation technique and Ketamine intravenous injection

Group 4: Control group

*F* F for One Way ANOVA test, pairwise comparison between each 2 groups were done using Post Hoc Test (Tukey)

p: *p* value for comparing between the studied four groups

p_1_: *p* value for comparing between Group 1 and Group 2

p_2_: *p* value for comparing between Group 1 and Group 3

p_3_: *p* value for comparing between Group 1 and Group 4

p_4_: *p* value for comparing between Group 2 and Group 3

p_5_: *p* value for comparing between Group 2 and Group 4

p_6_: *p* value for comparing between Group 3 and Group 4

^a^Statistically significant with control

^c^Statistically significant at *p ≤* 0.05

^d^*m* minutes

^e^*H* hours

**Fig. 3:**
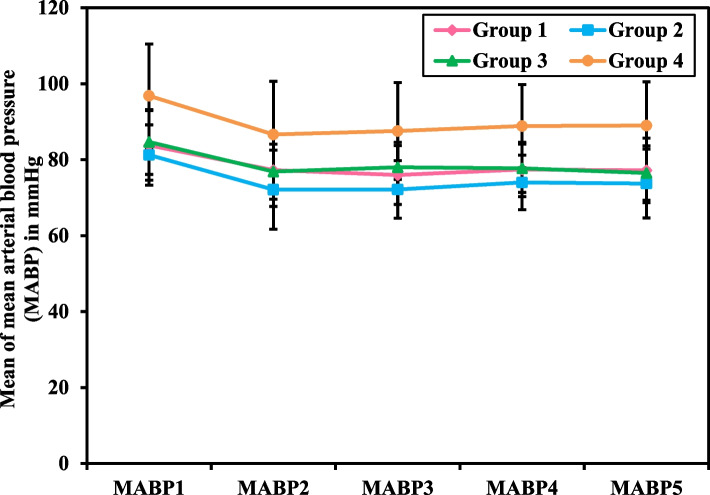
Intra-Operative Mean Arterial Blood Pressure (MABP) Measurements of Female Patients Undergoing Gynecological Laparoscopic Surgery

**Fig. 4:**
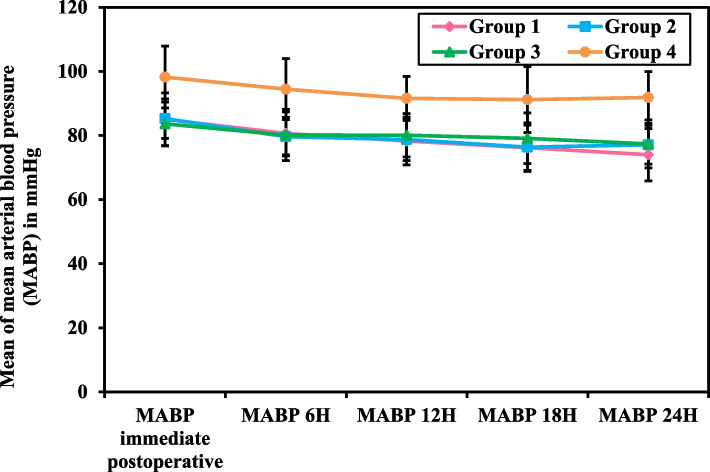
Post-Operative Mean Arterial Blood Pressure (MABP) Measurements of Female Patients Undergoing Gynecological Laparoscopic Surgery

In (Table 3), (Figs. 3 and 4), the Mean Arterial Blood Pressure measurement recorded every 15 minutes intraoperatively (MABP 1 -5) and those measured immediate postoperative (MABP IP) till 24 hours postoperative (MABP 24h) was significantly less in groups 1, 2 and 3compared to group 4 (*p<*0.001). At the same time, there was no significant difference between each intervention group and the other regarding the percentage decrease in MABP 1 -5. While MABP IP to 24h postoperative was higher in all the intervention groups with no statistically significant difference, except for group 1 compared to group 4, which was found to be statistically significant (12.28 ± 11.77 and 6.10 ± 7.24, *p=*0.025).

**Table 3:** Intra- and post-operative Mean Arterial Blood Pressure (MABP) measurements of female patients undergoing gynecological laparoscopic surgery

**Mean arterial blood pressure (MABP) in mmHg**	**Group 1** **(No. = 36)**	**Group 2** **(No. = 36)**	**Group 3** **(No. = 36)**	**Group 4** **(No. = 36)**	**F**	*p*
**Intra-operative MABP**						
**MABP1 (Basal MABP)**	83.78^a^ ± 9.16	81.25^a^ ± 7.94	84.67^a^ ± 8.54	96.83 ± 13.63	17.162^b^	<0.001^b^
**MABP2 (after 15 m.**^**c**^**)**	77.25^a^ ± 9.55	72.11^a^ ± 10.42	76.86^a^ ± 7.24	86.64 ± 13.99	11.892^b^	<0.001^b^
**MABP3 (after 30 m.)**	75.97^a^ ± 7.76	72.17^a^ ± 7.60	78.00^a^ ± 6.57	87.56 ± 12.77	19.066^b^	<0.001^b^
**MABP4 (after 45 m.)**	77.42^a^ ± 7.15	74.00^a^ ± 7.20	77.72^a^ ± 6.35	88.86 ± 10.94	22.885^b^	<0.001^b^
**MABP5 (after 60 m.)**	77.19^a^ ± 8.50	73.72^a^ ± 9.06	76.50^a^ ± 7.14	89.00 ± 11.49	19.538^b^	<0.001^b^
**Percent Decrease from MABP 1 – 5**	**7.37 ± 9.65**	**9.13 ± 8.10**	**9.07 ± 9.96**	**7.61 ± 8.48**	**0.383**	**0.766**
**Significance between groups**	**p**_**1**_**=0.843, p**_**2**_**=0.856, p**_**3**_**=0.999, p**_**4**_**=1.000, p**_**5**_**=0.893, p**_**6**_**=0.904**					
**MABP from Immediate Postoperative (IP) till after 24 hours**						
**MABP IP**	85.03^a^ ± 8.27	85.28^a^ ± 6.14	83.64^a^ ± 6.80	98.22 ± 9.63	27.374^b^	<0.001^b^
**MABP 6H**^**d**^	80.64^a^ ± 6.71	79.64^a^ ± 6.02	80.14^a^ ± 8.01	94.44 ± 9.52	31.290^b^	<0.001^b^
**MABP 12H**	78.28^a^ ± 7.43	78.72^a^ ± 6.55	80.08^a^ ± 6.78	91.58 ± 6.85	30.153^b^	<0.001^b^
**MABP 18H**	76.19^a^ ± 7.04	76.36^a^ ± 7.55	79.11^a^ ± 7.92	91.19 ± 10.28	26.509^b^	<0.001^b^
**MABP 24H**	74.00^a^ ± 8.15	77.11^a^ ± 6.05	77.36^a^ ± 7.49	91.86 ± 8.10	41.020^b^	<0.001^b^
**Percent Decrease from MABP IPO – 24H**	**12.28 ± 11.77**	**9.29 ± 7.95**	**7.19 ± 9.05**	**6.10 ± 7.24**	3.171^b^	0.026^b^
**Significance between groups**	**p**_**1**_**=0.509, p**_**2**_**=0.091, p**_**3**_**=0.025**^**b**^**, p**_**4**_**=0.767, p**_**5**_**=0.456, p**_**6**_**=0.958**					

Data was expressed in mean ± SD

Group 1: Relaxation technique (breathing and muscle contraction/relaxation exercises)

Group 2: Ketamine intravenous injection

Group 3: Relaxation technique and Ketamine intravenous injection

Group 4: Control group

*F* F for One Way ANOVA test, pairwise comparison bet. each 2 groups were done using Post Hoc Test (Tukey)

p: *p* value for comparing between the studied four groups

p_1_: *p* value for comparing between Group 1 and Group 2

p_2_: *p* value for comparing between Group 1 and Group 3

p_3_: *p* value for comparing between Group 1 and Group 4

p_4_: *p* value for comparing between Group 2 and Group 3

p_5_: *p* value for comparing between Group 2 and Group 4

p_6_: *p* value for comparing between Group 3 and Group 4

^a^Statistically significant with control

^b^Statistically significant at *p ≤* 0.05

^c^*m* minutes

^d^*H* hours

**Fig. 5:**
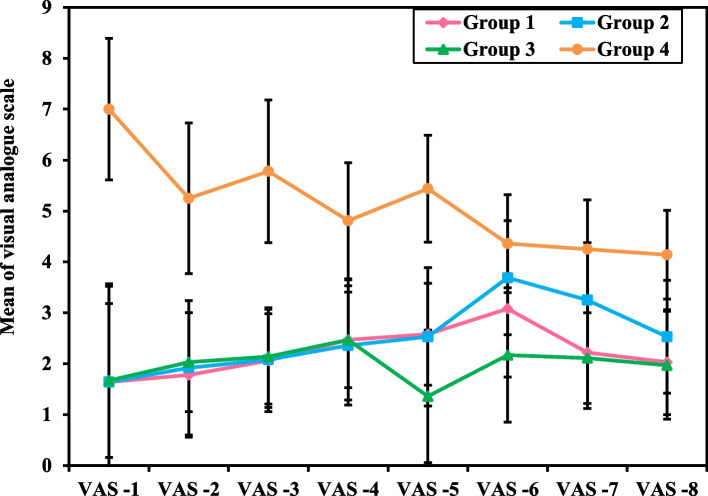
Visual Analogue Scale Measurements of Female Patients Undergoing Gynecological Laparoscopic Surgery

According to (Table 4 and Fig. 5), the Visual Analogue Scale measurements (VAS 1-8) were significantly less in the intervention groups 1, 2 and 3 compared to group 4, with a significant difference in the percentage increase of VAS 1 – 8 in the three intervention groups 1, 2 and 3 (24.71 ± 67.05, 32.17 ± 87.27 and 16.60 ± 46.61) compared to group 4 (-37.48 ± 22.31, *p<*0.001). The pain scores (VAS) increase occurred late in the intervention groups (1, 2, and 3) after VAS 6, while the VAS in group 4 was higher immediately postoperative.

**Table 4:** Visual analogue scale measurements of female patients undergoing gynecological laparoscopic surgery

**Visual analogue scale (VAS)**	**Group 1** **(No. = 36)**	**Group 2** **(No. = 36)**	**Group 3** **(No. = 36)**	**Group 4** **(No. = 36)**	**H**	*p*
**VAS -1 (immediately post-operative)**						
Mean ± SD.	1.64^a^ ± 1.88	1.64^a^ ± 1.93	1.67^a^ ± 1.51	7.0 ± 1.39	77.743^b^	<0.001^b^
Median (Min. – Max.)	1 (0 – 9)	1 (0 – 6)	2 (0 – 5)	7 (5 – 9)		
**VAS -2 (1 H**^**c**^** PO**^**d**^**)**						
Mean ± SD.	1.78^a^ ± 1.22	1.92^a^ ± 1.32	2.03^a^ ± 0.97	5.25 ± 1.48	73.003^b^	<0.001^b^
Median (Min. – Max.)	1.50(0 – 4)	2 (0 – 4)	2 (0 – 4)	5 (3 – 8)		
**VAS -3 (2H PO)**						
Mean ± SD.	2.06^a^ ± 0.92	2.08^a^ ± 1.02	2.14^a^ ± 0.93	5.78 ± 1.40	79.501^b^	<0.001^b^
Median (Min. – Max.)	2 (1 – 4)	2 (0 – 4)	2 (0 – 4)	6 (3 – 8)		
**VAS -4 (3H PO)**						
Mean ± SD.	2.47^a^ ± 0.94	2.36^a^ ± 1.17	2.47^a^ ± 1.18	4.81 ± 1.14	63.022^b^	<0.001^b^
Median (Min. – Max.)	2 (1 – 4)	2 (1 – 6)	3 (0 – 4)	5 (3 – 7)		
**VAS -5 (4H PO)**						
Mean ± SD.	2.58^a^ ± 1.0	2.53^a^ ± 1.36	1.36^a^ ± 1.30	5.44 ± 1.05	68.903^b^	<0.001^b^
Median (Min. – Max.)	2 (1 – 5)	2 (1 – 6)	2.5 (0 – 4)	6 (4 – 7)		
**VAS -6 (10H PO)**						
Mean ± SD.	3.08^a^ ± 1.34	3.69^a^ ± 1.12	2.17^a^ ± 1.32	4.36 ± 0.96	49.707^b^	<0.001^b^
Median (Min. – Max.)	3 (1 – 6)	4 (2 – 6)	2 (0 – 6)	4 (3 – 6)		
**VAS -7 (16 H PO)**						
Mean ± SD.	2.22^a^ ± 1.10	3.25^a^ ± 1.13	2.11^a^ ± 0.89	4.25 ± 0.97	65.201^b^	<0.001^b^
Median (Min. – Max.)	2 (1 – 6)	3 (1 – 5)	2 (0 – 3)	4 (3 – 6)		
**VAS -8 (24H PO)**						
Mean ± SD.	2.03^a^ ± 1.03	2.53^a^ ± 1.11	1.97^a^ ± 1.06	4.14 ± 0.87	60.126^b^	<0.001^b^
Median (Min. – Max.)	2 (1 – 5)	2 (1 – 5)	2 (0 – 4)	4 (3 – 6)		
**Percent Increase from VAS 1 – 8**	**24.71 ± 67.05**	**32.17 ± 87.27**	**16.60 ± 46.61**	**-37.48 ± 22.31**	**29.642**^**b**^	**<0.001**^**b**^
	**p**_**1**_**=0.971,p**_**2**_**=0.966,p**_**3**_**<0.001**^**b**^**,p**_**4**_**=0.997,p**_**5**_**<0.001**^**b**^**,p**_**6**_**<0.001**^**b**^					

Group 1: Relaxation technique (breathing and muscle contraction/relaxation exercises)

Group 2: Ketamine intravenous injection

Group 3: Relaxation technique and Ketamine intravenous injection

Group 4: Control group

*SD* Standard deviation, *H* H for Kruskal Wallis test, pairwise comparison bet. each 2 groups were done using Post Hoc Test (Dunn's for multiple comparisons test)

p: *p* value for comparing between the studied four groups.

p_1_: *p* value for comparing between Group 1 and Group 2

p_2_: *p* value for comparing between Group 1 and Group 3

p_3_: *p* value for comparing between Group 1 and Group 4

p_4_: *p* value for comparing between Group 2 and Group 3

p_5_: *p* value for comparing between Group 2 and Group 4

p_6_: *p* value for comparing between Group 3 and Group 4

^a^Statistically with control

^b^Statistically significant at *p ≤* 0.05

^c^*H* hours

^d^*PO* post-operative

Table 5 illustrates Riker sedation–agitation scores, where none of the patients was unarousable, one patient (2.8%) from group 3 was very sedated, 13.9% of group 1, 16.7% of group 2, 19.4% of group 3 were sedated. Meanwhile, 80.6% of Groups 1 and 2 and 77.8% of Group 3 were calm and cooperative, significantly higher than the control group (16.7%, *p<*0.001). Agitation was found in 5.6% of Group 1, 2.8% of Group 2, and none of Group 3, while 58.3% of the control group were agitated, and 25.0% of Group 4 were very agitated. Overall, 94.4% of Group 1 was non-agitated, 97.2 % of Group 2, and 100.0% of Group 3, which was found to be significantly higher than Group 4(16.7%, *p<*0.001), with no significant difference between each intervention group and the other. Meanwhile, the agitation was 5.6%, 2.8%, and 0.0% in groups 1,2 and 3, respectively, significantly lower than in group 4 (83.3%). Group 1 was 85 times more likely to be non-agitated (85 (15.938 – 453.307), *p<*0.001), group 2 was 175 times more likely to be non-agitated (175 (19.932–1536.448), *p<*0.001) and group 3 was protected against agitation.

**Table 5:** Riker sedation–agitation scores measurements of female patients undergoing gynecological laparoscopic surgery

	**Group 1** **(*n =* 36)**		**Group 2** **(*n =* 36)**		**Group 3** **(*n =* 36)**		**Group 4** **(*n =* 36)**		**χ**^**2**^	*p*
	**No.**	**%**	**No.**	**%**	**No.**	**%**	**No.**	**%**		
**Riker's = Emergence Agitation (EA)**										
1 Unarousable	0	0.0	0	0.0	0	0.0	0	0.0	90.196^a^	^MC^p <0.001^a^
2 Very sedated	0	0.0	0	0.0	1	2.8	0	0.0		
3 Sedated	5	13.9	6	16.7	7	19.4	0	0.0		
4 Calm and co-operative	29	80.6	29	80.6	28	77.8	6	16.7		
5 Agitated	2	5.6	1	2.8	0	0.0	21	58.3		
6 Very agitated	0	0.0	0	0.0	0	0.0	9	25.0		
7 Dangerous agitation	0	0.0	0	0.0	0	0.0	0	0.0		
< 4= Non-agitated	34	94.4	35	97.2	36	100.0	6	16.7	99.499^a^	<0.001^a^
≥ 4= Agitated ®	2	5.6	1	2.8	0	0.0	30	83.3		
**OR (LL – UL 95% C.I)**	**85 (15.938 – 453.307)**		**175 (19.932–1536.448)**		**–**					
***p***	**<0.001**^**a**^		**<0.001**^**a**^		**–**					

Group 1: Relaxation technique (breathing and muscle contraction/relaxation exercises)

Group 2: Ketamine intravenous injection

Group 3: Relaxation technique and Ketamine intravenous injection

Group 4: Control group

*χ*^*2*^ Chi square test, *MC* Monte Carlo, *OR* Odd`s ratio, *C.I* Confidence interval, *LL* Lower limit, *UL* Upper Limit, *p*: *p* value for comparing between the studied four groups

^a^Statistically significant at *p ≤* 0.05

97.2% of groups 1, 2, and 3, with 83.3% of group 4, had no postoperative complications. Nausea appeared in only one patient in each intervention group and 6 patients of the controls, significantly less than in group 4 (MC*p=* 0.033); however, the intergroup differences were not statistically significant. Hemodynamic instability occurred in 25.0%, 13.9%, and 19.4% in groups 1,2 and 3, respectively, significantly less than in group 4 (66.7%), *p<*0.001. Recovery time, according to Modified Aldert's score, was significantly less in group 3 compared to the control (8.0 ± 2.53, 7.56 ± 3.07, and 7.56 ± 3.07 minutes respectively, while it was less than the controls in group 2 (9.83 ± 3.22) but did not reach the significant level, *p ≤* 0.05 (Table 6).

**Table 6:** Post-operative complications and recovery time (according to modified aldert’s score) of female patients undergoing gynecological laparoscopic surgery

**Post-operative complications**	**Group 1** **(*n =* 36)**		**Group 2** **(*n =* 36)**		**Group 3** **(*n =* 36)**			**Group 4** **(*n =* 36)**		**χ**^**2**^	*p*
	**No.**	**%**	**No.**	**%**	**No.**		**%**	**No.**	**%**		
**Gastrointestinal complications**											
None	35	97.2	35	97.2	35		97.2	30	83.3	6.511^b^	^MC^*p=* 0.033^b^
Nausea	1	2.8	1	2.8	1		2.8	6	16.7		
**Significance between groups**	^**FE**^**p**_**1**_**=1.000,**^**FE**^**p**_**2**_**=1.000,**^**FE**^**p**_**3**_**=0.107, p**_**4**_^**FE**^**p**_**1**_**=1.000,**^**FE**^**p**_**5**_**=0.107, p**_**6**_^**FE**^**=0.107**										
**Hemodynamic instability**											
No	27	75.0	31	86.1	29		80.6	12	33.3	29.059^b^	<0.001^b^
Yes	9	25.0	5	13.9	7		19.4	24	66.7		
**Significance between groups**	**p**_**1**_**=0.234, p**_**2**_**=0.571, p**_**3**_**<0.001**^**b**^**, p**_**4**_**=0.527, p**_**5**_**<0.001**^**b**^**, p**_**6**_**<0.001**^**b**^										
**Recovery time according to Modified Aldert’s score in minutes**											
Mean ± SD.	8.0^a^ ± 2.53			9.83 ± 3.22		7.56^a^ ± 3.07		10.69 ± 3.25		21.864^b^	<0.001^b^
Median (Min. – Max.)	8 (4 – 5)			10 (4 –15)		7 (3 – 15)		10 (5 –15)			

Group 1: Relaxation technique (breathing and muscle contraction/relaxation exercises)

Group 2: Ketamine intravenous injection

Group 3: Relaxation technique and Ketamine intravenous injection

Group 4: Control group

*SD* Standard deviation, *H* H for Kruskal Wallis test, pairwise comparison bet. each 2 groups were done using Post Hoc Test (Dunn's for multiple comparisons test)

p: *p* value for comparing between the studied four groups

p_1_: *p* value for comparing between Group 1 and Group 2

p_2_: *p* value for comparing between Group 1 and Group 3

p_3_: *p* value for comparing between Group 1 and Group 4

p_4_: *p* value for comparing between Group 2 and Group 3

p_5_: *p* value for comparing between Group 2 and Group 4

p_6_: *p* value for comparing between Group 3 and Group 4

^a^Statistically with control

^b^Statistically significant at *p ≤* 0.05

## Discussion

EA is a known and not uncommon phenomenon that occurs during early postoperative awareness after general anesthesia. Patients who have post-traumatic stress disorder (PTSD) have shown higher incidence as well as more resistant symptoms of EA due to altered neurotransmitter system [5].

Trauma could be experienced and perceived differently [1]; with recent massive influential events such as the COVID-19 pandemic [27, 28] and wars around the world, specifically the Middle East [29, 30], it is more likely to expect PTSD. Accordingly, preparing a reliable anesthetic protocol would be crucial to ensuring smooth recovery and emergence [1]. Variable interventions were introduced pre-, intra-, and post-operatively to avoid EA in PTSD patients, with the less invasive, aversive, and lower harmful side effects being the most favorable [1, 5].

In the current study, we aimed to compare three different intervention techniques used for avoiding EA in PTSD patients undergoing gynecological laparoscopic surgery. The first strategy was applying preoperative relaxation techniques (deep breathing exercises and PMR); the second was administering intraoperative intravenous Ketamine injection; and the third was combining both previously mentioned strategies. All three intervention groups (1, 2, and 3) showed significantly lower HR 1-5 and HR IP to 24h than the control group 4. Group 1 (relaxation technique group) showed lower HR 1-5 and IP to 24h than the other two intervention groups. That could be attributed to the vagal stimulation of relaxation techniques on HR [31]. Percentage decrease of HR from the first intra-operative measurement (HR1) consistently till the 5th (HR5) was significantly lower in the three intervention groups compared to control.

In contrast, the percentage decrease from HR IP to 24h was higher in group 2 (intraoperative ketamine group); the higher HR IP could explain that in group 2 due to the effect of ketamine on increasing the HR (sympathomimetic effect) [32, 33] in addition to the stress of extubation and recovery from anesthesia. After 6 hours from the operation (HR 6h), the HR began to decrease gradually till HR 24h with fading of the ketamine effect [34], leading to a higher percentage decrease in this group. On the other hand, in group 3 (combined relaxation technique/intra-operative ketamine group), the relaxation techniques minimized the sympathomimetic effect of ketamine on HR IP, allowing the HR decrease to fall with fewer intervals till HR 24h, hence leading to smaller percentage decrease of HR IP-HR 24h.

Recently, non-pharmacological interventions have gained popularity, mainly because they yield effective results regarding smooth recovery, early ambulation, and hospital discharge at less cost [35, 36]. In light of these findings, Barabady et al. (2020) in Iran concluded that relaxation techniques based on deep breathing and muscle relaxation (Benson's deep relaxation) reduced heart rate and blood pressure and stabilized patients' respiration [35]. Likewise, Salah et al. (2022) in Egypt confirmed the efficacy of deep breathing exercises in pain reduction and hemodynamic stability (including heart rate and blood pressure) of patients during chest tube removal following cardiac surgery [37]. In agreement, Pardede et al. (2020) in Indonesia found that deep breath relaxation and lavender aromatherapy were effective7 in reducing preoperative anxiety, heart rate, and anticipated pain [14].

The findings of our study regarding the ketamine effect were supported by Demir et al. (2018) in Turkey, who proved the effectiveness of a sub-anesthetic dose of ketamine in reducing HR, blood pressure, pain, and EA [38]. Also consistent with our results, Andibirku et al. (2022) in Ethiopia reported a significant rise followed by a decrease in HR after 10 minutes in the ketamine group, which did not occur in the comparative group who received thiopental in addition to ketamine [39].

In the current work, the three intervention groups (1, 2, and 3) generally showed significantly lower MABP 1-5 and MABP IP to 24h than the control group 4. However, intra-operatively, MABP 1-5 percentage decrease showed no significant difference between the four groups. That could be due to the effect of other anesthetic drugs, which were given equally in all groups, including the control one, such as intravenous propofol administered during induction and inhalational isoflurane used for maintenance of anesthesia. Interestingly, only group 1 (relaxation technique group) MABP IP to 24h percentage decrease was statistically significant, while the other intervention groups, 2 (intraoperative ketamine group) and 3 (combined relaxation technique/intra-operative ketamine group), did not show a significant difference. As mentioned before, ketamine is a sympathomimetic drug, which causes vasoconstriction and hence higher blood pressure and, therefore, a smaller percentage decrease in MABP IP to 24h. On the other hand, relaxation techniques are parasympathomimetic and cause vasodilatation and vagal stimulation, which allowed a percentage decrease in MABP IP to 24h, especially when unopposed with ketamine (in group 1). At the same time, it was opposed to ketamine (in group 3).

Concordant results were reported by Barabady et al. (2020) in Iran [35] and Salah et al. (2022) in Egypt [37] concerning the relaxation techniques effect on the MABP in our study. In addition, Ali et al. (2022) in Iraq similarly concluded that the heart rate and mean atrial blood pressure were initially higher in the group of patients who received ketamine in addition to propofol than in the group with propofol alone. However, the difference was not statistically significant. Yet, the ketamine/propofol mixture was significantly better for maintaining hemodynamic stability for 30 minutes after induction [40].

The present study showed an increase in the pain scores (VAS) that occurred late in the intervention groups (1, 2, and 3) after VAS 6 because of the effect of relaxation exercises, ketamine, or both, which controlled the pain with their early application. The VAS in group 4 was higher immediately postoperative (since VAS 1) because the pain was not interfered with. That's to say, the first rescue analgesia was needed late post-operatively among the intervention groups (1, 2, and 3), while it was necessary from the start of the postoperative period in group 4.

Supportive findings were concluded by Baljon et al. (2022) in Saudi Arabia [41], who found breathing exercises to help lower VAS scores. However, they used breathing exercises combined with foot reflexology and massage (BRM) during labor. Salah et al. (2022) in Egypt [37] and Pardede et al. (2020) in Indonesia [14] also agreed on the effect of deep breathing on pain reduction after surgery, while Chaudhuri et al. (2020) in India proved that progressive muscle relaxation exercise was highly effective in reducing anxiety in painful conditions (such as coronary artery disease) [42].

The results of our study concerning the ketamine effect on the VAS score were consistent with the findings of Demir et al. (2018) in Turkey [38] and Han et (2022) in China, who confirmed the effectiveness of ketamine in decreasing postoperative VAS score, nausea, and vomiting and achieving better recovery from anesthesia [43].

In this study, participants in the intervention groups (1, 2, and 3) were significantly calmer, more cooperative, and less agitated than group 4. Cases in Group 1 were 85 times more likely to be non-agitated, those in Group 2 were 175 times more likely, and all of Group 3 were protected against agitation. Complete protection among group 3 cases could be due to the additive effect of parasympathetic stimulation of the relaxation techniques and the analgesic effect of ketamine.

No study measured the effect of relaxation techniques on EA specifically to our knowledge. However, lower anxiety and pain, as well as higher stability of patients who were offered relaxation techniques, are in favor of this conclusion. Likewise, Elsayed et al. (2020) in Egypt [44] found progressive muscle relaxation to be significantly effective in decreasing postoperative pain and improving the postoperative quality of patients' recovery after surgery.

The role of ketamine in decreasing the risk and symptoms of EA in our work was supported by the Yan et al. (2015) meta-analysis [45] and Demir et al. (2018) in Turkey [38]. Concomitantly, Lovestrand et al. (2017) in the USA [5] and Closson et al. (2024) in the USA recommended a sub-dose of ketamine early intraoperatively during the general anesthesia clinical practice guidelines for patients with PTSD [46].

Furthermore, the current work findings showed that postoperative nausea and hemodynamic instability were significantly less in all three intervention groups compared to the control group with no statistically significant intergroup difference, which could also be explained by the equal effectiveness of both preoperative relaxation techniques and early intra-operative intravenous ketamine in stabilizing the patient post-operatively (regarding nausea and hemodynamics) without a statistically significant effect on the recovery time. Concordantly, Ibrahim et al. (2020) in Egypt [47] and Dewi et al. (2021) in Indonesia [48] concluded that postoperative nausea and vomiting significantly decreased after diaphragmatic (deep) breathing performance. The results of Yan et al. (2015) meta-analysis [45], Demir et al. (2018) in Turkey [38], and Han et al. (2022) in China [43] were also in agreement with our findings regarding the ketamine effectiveness in these aspects.

As observed from the results of our study, the effect of intra-operative low-dose Ketamine injection in decreasing the incidence of EA was confirmed in patients with PTSD. Preoperative relaxation techniques (breathing exercises and PMR) had similar effects on lowering HR, MABP, VAS score, and EA without much privilege of their combination. Relaxation techniques could reduce costs by sparing anesthetic and analgesic drugs and minimizing hospitalization time. Accordingly, we suggest that relaxation techniques (namely, breathing exercises and PMR) could be included in the preoperative preparation protocol to decrease the risk and/or symptoms of EA, particularly among patients who test positive for PTSD.

## Strengths and limitation

This study is the first to assess and contrast the effects of ketamine and breathing exercises on EA among PTSD women. A randomized, double-blinded design was utilized to minimize potential biases. Nevertheless, our study was limited to a follow-up period of 24 hours; more clinical trials with more extended follow-up periods are warranted to explore the long-term benefits of these interventions among PTSD patients.

## Conclusion

Preoperative relaxation techniques (breathing exercises and PMR) significantly lowered HR, MABP, VAS score, and EA than controls. These effects were not significantly different from intra-operative ketamine injection or the combination of both (relaxation techniques and ketamine). We recommend routine preoperative screening for PTSD and the application of relaxation techniques (breathing exercises and PMR) in the preoperative preparation protocol of PTSD-positive cases. Further studies on using preoperative relaxation techniques in general could be cost-effective.

## Data Availability

Data is available upon request to the corresponding author.
